# Deep Learning-Based Protein Half-Life Prediction for Identifying Rate-Limiting Enzymes in Metabolic Pathways to Alleviate Bottleneck Reactions

**DOI:** 10.4014/jmb.2601.01071

**Published:** 2026-04-27

**Authors:** Yunhyeok Lee, Jun Ren, Jingyu Lee, Minh Thi-Hong Tran, Yubin Kim, Youngseo Chang, So Hee Oh, Hyang-Mi Lee, Dokyun Na

**Affiliations:** Department of Biomedical Engineering, Chung-Ang University, Seoul 06974, Republic of Korea

**Keywords:** Protein half-life, Machine learning, Metabolic engineering, Metabolic pathway, Lycopene

## Abstract

In synthetic metabolic pathways, the intracellular level of enzymes is a critical determinant of pathway efficiency and, thus, short-lived enzymes create bottlenecks and limit overall metabolic productivity due to their low abundance. However, since studies on protein half-life remain limited in bacteria, its accurate prediction is a significant challenge. To address this, we developed a machine learning model, ProHL, for the classification of short-lived and long-lived proteins. ProHL employs a multimodal strategy, integrating ProteinBERT encodings (at both residue and sequence levels) with physicochemical encodings of the protein sequences. This integration enables the effective capture of both local and global sequence features, thereby ensuring accurate half-life classification. When evaluated on an independent test dataset of *E. coli* proteins, ProHL achieved an accuracy of 0.818 and a Matthew's correlation coefficient of 0.624. To demonstrate its practical utility in metabolic engineering, we classified CrtE, CrtB, and CrtI enzymes involved in lycopene biosynthesis and identified that only CrtB as short-lived. Consistent with this prediction, when CrtB was additionally expressed in a lycopene-producing base strain, lycopene production in *E. coli* increased up to 25%. Our computational framework, ProHL, identifies short-lived, rate-limiting enzymes by employing *in silico* prediction of enzyme half-life. This approach provides a viable strategy for alleviating metabolic bottlenecks, ultimately enhancing metabolic productivity.

## Introduction

**Fig. 1:**
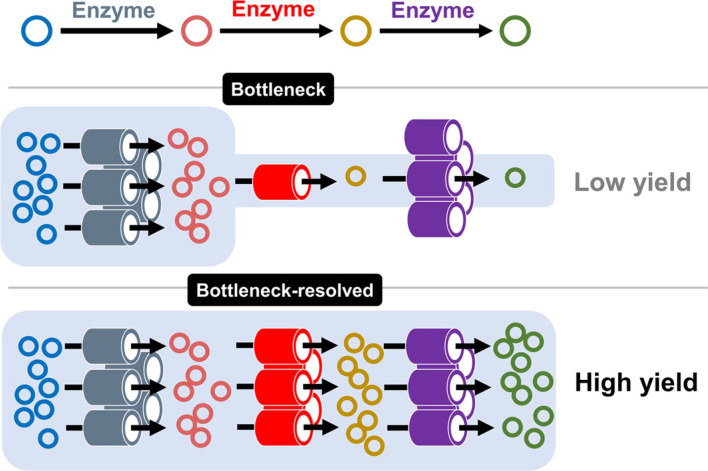
Bottleneck in a synthetic metabolic pathway due to short life of enzyme. Short-lived enzyme creates a rate-limiting step that acts as bottleneck in synthetic pathways. Reinforcing the expression of the bottleneck enzyme increases their intracellular abundance, thereby alleviating rate limit and enhancing overall metabolite production.

In metabolic engineering, non-inherent compounds can be produced in bacteria by introducing synthetic metabolic pathways composed of recombinant enzymes [1, 2]. The intracellular level of recombinant enzymes is one of the critical factors that influence the efficiency of synthetic metabolic pathways. Lowly expressed enzymes, in particular, often act as a bottleneck in the pathway, which results in inefficient substrate-to-product conversion [3, 4] (Fig. 1). Furthermore, the resulting accumulation of intermediate metabolites may cause stress or toxicity to the host strain [5-7]. Thus, it is important to maintain optimally balanced levels of the recombinant enzymes to avoid accumulation of intermediate metabolites as well as to achieve high production yields [8].

To date, experimental and computational strategies have focused on designing genetic sequences such as promoters and translation initiation regions that modulating transcription and translation to achieve desired levels of enzymes [9-11]. In addition to transcription and translation, however, enzyme proteostasis - governed by enzyme half-life - is also a critical factor that influences the efficiency of synthetic metabolic pathways; short-lived enzymes may act as a bottleneck in the pathway [5]. Thus, enzymes at low intracellular levels due to their short half-life decrease overall production yield. However, there have been limited studies on protein half-life prediction and, thus, identification of rate-limiting enzymes in a pathway for enhanced metabolic efficiency is a significant challenge.

Several experimental methods to determine protein half-life have been established. However, these approaches are technically demanding and time-consuming, making it difficult to identify short-lived bottleneck enzymes within synthetic metabolic pathways. This challenge can hinder progress in metabolic engineering, which is increasingly shifting from *ad hoc* engineering to the rational design of microbial cell factories [12, 13]. Therefore, there is an increasing demand for predictive methods to facilitate the rapid identification of rate-limiting enzymes in synthetic pathways for industrial applications [14].

A commonly used method to predict the half-life of proteins in bacteria is the N-end rule [15]. However, this method is limited because not all proteins are processed to expose N-terminal residues, and additional degrons have been identified at the C-terminus as well as within internal regions [16-19]. Recent studies further show that protein half-life is influenced by various physicochemical properties [20], indicating that N-end rule-based predictions are often inaccurate. For example, proteins such as DcuR, BioD, and Rph in *E. coli*, were predicted to have half-lives > 10 hours based on the N-end rule, whereas experimental values ranged from 0 to 5 minutes [21]. Thus, more comprehensive prediction models that incorporate multiple protein features are needed.

With advances in machine learning algorithms, computational models have increasingly been employed to handle diverse biological features across different modalities [22]. However, due to experimental difficulty and inconsistent measurement conditions, no standardized dataset has been available to sufficiently train predictive models. Despite these challenges, the accumulation of half-life data in eukaryotes has enabled the development of several machine learning-based prediction models, including those trained on human and mouse datasets by Tan *et al*. [23], PlifePred using mammalian peptide data [14], and PLTNUM based on *Mus musculus* NIH3T3 fibroblast protein data [24]. In contrast, due to the limited availability of sufficient half-life measurements in bacteria, no bacterial protein half-life prediction models have been reported to date. Recently, a proteome-wide, standardized dataset of protein half-life in *E. coli* was generated through high-throughput experimental measurements [25]. Although the dataset remains smaller than those in other fields, its standardization now provides an opportunity to develop a machine learning model for bacterial protein half-life prediction.

**Fig. 2:**
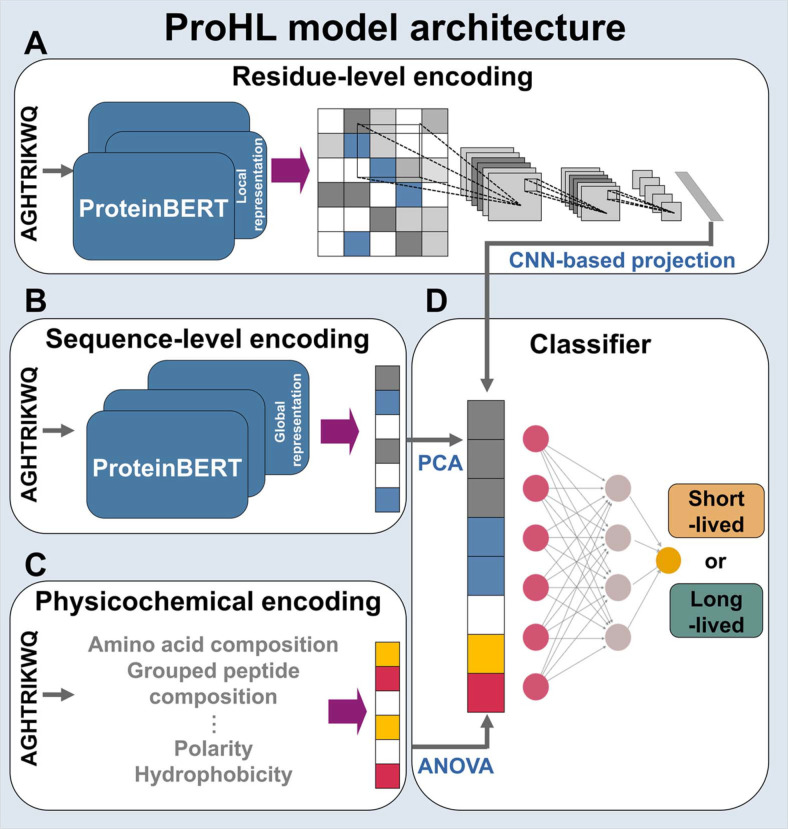
Architecture of protein half-life prediction model. The model integrates three feature types: (**A**) residue-level and (**B**) sequence-level encodings calculated using ProteinBERT and (**C**) physicochemical encodings calculated using iFeature and Pfeature. Residue-level encodings are processed through CNN to extract local contextual information and convert to 1D encoding for concatenating with other encodings. The concatenated encodings are fed into (**D**) the fully connected neural network for classification.

In this study, we develop a machine learning classification model, ProHL, that categorizes bacterial proteins into short- and long-lived groups according to their half-lives (Fig. 2). Instead of directly measuring enzyme stability, we demonstrated a proof-of-concept application by modifying the expression of a predicted short-lived enzyme in a metabolic engineering context. As a practical demonstration of its applicability in metabolic engineering, we applied ProHL to identify a short-lived enzyme within the lycopene biosynthetic pathway introduced in *E. coli*. This pathway comprises three recombinant enzymes: phytoene synthase (CrtB), geranylgeranyl diphosphate synthase (CrtE), and phytoene desaturase (CrtI), which convert farnesyl diphosphate (FPP) to lycopene [26]. ProHL predicted CrtB to be short-lived, and additional expression of CrtB in a lycopene-producing *E. coli* significantly increased lycopene production. This result indicates that CrtB functions as a rate-limiting enzyme due to its short half-life and demonstrates that ProHL can effectively identify bottleneck enzymes in synthetic metabolic pathways.

## Materials and Methods

### Data Preparation and Preprocessing

A total of 1,148 *E. coli* protein half-life data were compiled from a proteome-wide study in which protein half-lives were experimentally measured [25], and this dataset was used for model training. For independent evaluation, we additionally collected 11 *E. coli* protein half-life data obtained via Western blot or immunofluorescence, which were not included in the training dataset [21, 27, 28]. In previous studies, the average half-life of *E. coli* proteins has been reported to be about 4-6 hours and about 10% of them are less than 60 min. It has been commonly considered that a half-life of less than 1 hour is short-lived [25, 29] and those short-lived proteins are typically characterized by high structural disorder and are regulatory factors that have a rapid turnover for dynamic control of cellular physiology [25, 29, 30]. Thus, in this study we defined the terms “short-lived” and “long-lived” based on the classification criteria of the ProHL model. Following the convention established in previous high-throughput protein turnover studies [25, 29], a half-life of 1 hour was utilized as the absolute threshold for stability categorization. Consequently, ProHL was trained as a binary classifier to identify proteins with a predicted half-life of less than 1 h.

Of the compiled proteins, peptides shorter than 50 amino acids in length were removed from the dataset, since short peptides may exhibit distinct properties from those of proteins. To reduce redundancy and prevent model overfitting to highly similar sequences, the remaining proteins were clustered using CD-HIT with a sequence identity cutoff of 0.5, and redundant sequences were removed [31]. As a result, the *E. coli* training dataset contained 1,118 proteins (1,035 long-lived and 83 short-lived) and the *E. coli* test dataset contained 11 proteins (7 long-lived and 4 short-lived).

### Feature Generation and Preprocessing

To convert protein sequences into numerical representations suitable for machine learning, we employed three encoding methods: ProteinBERT for residue-level and sequence-level encodings [32], and iFeature [33] and Pfeature [34] for physicochemical encodings. ProteinBERT is a pre-trained language model for protein sequences, trained on 106 million protein sequences and 45,000 Gene Ontology annotations, and designed to generate both residue-level and sequence-level protein encodings. Specifically, it generates contextualized residue-level encodings that capture the biochemical context of individual amino acid residues, as well as a sequence-level encoding that summarizes the overall properties of the protein sequence. iFeature and Pfeature are comprehensive toolkits for generating various physicochemical encodings derived from protein sequences.

To prevent overfitting, we reduced the dimensionality of the feature space to an appropriate level as follows, thereby simplifying the features while preserving essential information. The 2D residue-level encodings were converted into a 1D encoding through a convolutional neural network (CNN), and the parameters of the CNN were jointly optimized with those of the entire model. Specifically, global mean pooling is commonly employed to compress residue-level encodings—typically of size (embedding dimension × sequence length)—into a 1D format. However, recent studies indicate that CNNs more effectively capture local contextual representations within residue-level encodings compared with global mean pooling [35]. To leverage this advantage, ProHL transforms the 2D residue-level encoding into a 1D representation using a CNN architecture consisting of two consecutive Conv2D–AveragePooling2D blocks. Each block employs filters with a kernels, followed by dropout layers to mitigate overfitting, and these parameters were jointly optimized with the parameters of the entire model (see Model construction and optimization). Sequence-level encodings were reduced by principal component analysis (PCA), retaining the components that explained 95% of the variance [36], which is commonly applied in reducing language model encodings [37, 38]. Physicochemical encodings were selected based on statistical correlation analysis with respect to target classes [39]. Encoding (feature) values were standardized using z-score normalization to mitigate scale-induced biases [40]. Subsequently, an analysis of variance (ANOVA) was performed to evaluate the association between the target variable (protein half-life class) and each encoding. Encodings with a *p*-value > 0.01 were considered statistically insignificant and thereby discarded.

### Model Construction and Optimization

ProHL consists of three encoding modules and a classification module (Fig. 2). The concatenated encodings generated from the encoding modules are fed into a fully connected network for binary classification (classification module). To enhance the predictive performance of ProHL, we optimized eight key hyperparameters across the CNN for projecting a 2D residue-level encoding into a 1D encoding and the fully connected network for classification: kernel size, filters, layers, units, dropout, learning rate, max epoch, and batch size [41].

To determine the optimal hyperparameter configuration, we performed a systematic grid search coupled with 10-fold cross-validation on the training dataset. The parameter set yielding the highest mean Matthews Correlation Coefficient (MCC) across the folds was selected. A total of 1,296 candidate models were evaluated by varying the following parameters: (1) CNN architecture: kernel sizes (7 × 7, 9 × 9) and filter counts (8, 16); (2) Classification network: layer depths (1, 2, 3) and hidden unit sizes (512, 1024, or input encoding length); and (3) Training hyperparameters:dropout rates (0.1, 0.2), learning rates (10^-3^, 10^-5^), maximum epochs (10, 20, 30), and batch sizes (32, 64, 128). Model training was conducted using the Adam optimizer, incorporating an early stopping strategy (patience = 5) to mitigate overfitting. The kernel size determines the spatial dimensions of the 2D convolutional window, while the number of filters dictates the number of kernels applied to extract diverse local features from the input matrix. The count of layers and hidden units specify the depth and capacity of the fully connected network, thereby modulating the model’s representational power. Dropout denotes the fraction of neurons randomly masked during training to mitigate overfitting and enhance generalization. Furthermore, the learning rate, the number of epochs, and the batch size define the step size for gradient updates and the total training iterations, respectively; these parameters critically influence convergence stability and computational efficiency.

### Model Evaluation

Predictive performance was assessed in terms of the following metrics: accuracy (ACC), sensitivity (SEN), specificity (SPE), positive predictive value (PPV), negative predictive value (NPV), and MCC. These metrics were calculated based on classification results, quantified by the counts of true positives (TP), true negatives (TN), false positives (FP), and false negatives (FN).

$$\text{ACC}=\frac{\text{TP}+\text{TN}}{\text{TP}+\text{TN}+\text{FP}+\text{FN}}$$ (1)

$$\text{SEN}=\frac{\text{TP}}{\text{TP}+\text{FN}}$$ (2)

$$\text{SPE}=\frac{\text{TN}}{\text{TN}+\text{FP}}$$ (3)

$$\text{PPV}=\frac{\text{TP}}{\text{TP}+\text{FP}}$$ (4)

$$\text{NPV}=\frac{\text{TN}}{\text{TN}+\text{FN}}$$ (5)

$$\text{MCC}=\frac{\left(\text{TP}\times \text{TN})-(\text{FP}\times \text{FN}\right)}{\sqrt{\left(\text{TP}+\text{FP})\times (\text{TP}+\text{FN})\times (\text{TN}+\text{FP})\times (\text{TN}+\text{FN}\right)}}$$ (6)

where TP denotes the number of cases that are correctly predicted as long-lived, TN denotes the number of cases that are correctly predicted as short-lived.

### Implementation of ProHL Web Server

The ProHL model is available for public access at our web server. The ProHL web server was implemented using the Python-based Flask framework (version 2.2.5) running on Python 3.8.5. The system was developed and executed on Ubuntu 24.04.2. The web interface was designed using HTML5 and Bootstrap 5.3.0, enabling responsive rendering and a consistent user experience across modern web browsers.

### Genetic Engineering of Lycopene-Producing *E. coli* Strain

A previously engineered *E. coli* strain was used as the base strain for lycopene production in this study [26]. This strain harbors three genes of the lycopene biosynthetic pathway—*crtI*, *crtE*, and *crtB* from *Deinococcus wulumuqiensis* R12—which convert farnesyl diphosphate (FPP) into lycopene. In addition, four genes that enhance flux through the non-mevalonate (MEP) pathway—1-deoxy-D-xylulose-5-phosphate synthase (*dxs*), 1-deoxy-D-xylulose 5-phosphate reductoisomerase (*dxr*), isopentenyl diphosphate isomerase (*idi*), and farnesyl diphosphate synthase (*ispA*) from *E. coli*—were co-expressed to increase precursor supply.

To evaluate the effect of additional CrtB expression, which was predicted to be short-lived by the ProHL model, and to compare it with the effects of CrtI and CrtE, each gene was individually cloned under the control of the tac promoter into a plasmid with a p15A origin. These plasmids were separately introduced into the base lycopene-producing strain to generate the corresponding engineered strains. Cells were grown in LB broth (1% tryptone, 0.5% yeast extract, and 1% NaCl) at 30°C with 25 μg/mL of chloramphenicol, 100 μg/mL of ampicillin and 50 μg/mL kanamycin.

### Lycopene Measurement

A single colony was inoculated into 5 mL of LB medium supplemented with the appropriate antibiotics and incubated overnight. Subsequently, 0.5 mL of the seed culture was transferred into a 50 mL flask containing fresh LB medium and induced by 1% L-arabinose (Bio Basic, CAS#5328-37-0, Canada), and various concentrations of IPTG (0–1 mM; Bio Basic, CAS# 367-93-1, Canada). Cultures were then incubated at 30°C with shaking at 200 rpm for up to 16 h. All experiments were performed in the dark because lycopene is light-sensitive, and lycopene measurements were performed in triplicate.

For lycopene production, a dual-layer induction system was implemented. The basal pathway comprised four MEP pathway genes (*dxs*, *dxr*, *idi*, and *ispA*) under an L-arabinose–inducible promoter and three core carotenoid biosynthetic genes (*crtI*, *crtE*, and *crtB*) driven by a synthetic constitutive promoter (BBa_J23118). Induction with L-arabinose established a consistent precursor supply. For the targeted augmentation of specific pathway enzymes, an additional plasmid harboring either *crtB*, *crtI*, or *crtE* under the tac promoter was introduced. The expression of these supplemental genes was induced with various concentrations of IPTG.

Cell growth was monitored at 600 nm (OD_600_) using a Hitachi U5100 UV–Vis spectrophotometer (Japan). At 4 h, 8 h, 12 h, and 16 hours post-incubation, cells from 50 mL of culture were harvested by centrifugation at 7600 ×g for 5 min at 4°C. The cell pellets were washed once with distilled water, resuspended in acetone, and incubated at 55°C for 15 min to extract lycopene. After centrifugation (7,600 × g, 10 min, 25°C), the supernatant was analyzed by HPLC for lycopene quantification.

For HPLC analysis, 20 μL of each sample was injected into a ZORBAX Eclipse Plus C18 column (4.6 × 150 mm, 5 μm; Agilent, USA) and separated under isocratic conditions with a mobile phase consisting of 80% acetone, 15% methanol, and 5% isopropanol at a flow rate of 1 mL/min for 20 min at 30 C. Lycopene was detected at 472 nm, and commercially available lycopene (Sigma Aldrich, USA) was used as a standard. All experiments were conducted under dark conditions to prevent lycopene isomerization due to light exposure [42, 43].

## Results and Discussion

### Construction of Protein Half-Life Prediction Model (ProHL)

Protein half-life is influenced by multiple intrinsic and extrinsic determinants in proteins, including sequence-contextual patterns, structural disorder, and physicochemical properties [16-20]. To build a machine-learning model that accurately classifies proteins as long-lived or short-lived, these determinants must be incorporated during training (Fig. 2). In this study, we utilize ProteinBERT, iFeature, and Pfeature to generate protein encodings that enable the model to incorporate determinants relevant to protein half-life classification, from sequence-contextual patterns to physicochemical properties. ProteinBERT is a pre-trained language model for protein sequences, trained on 106 million protein sequences and 45,000 Gene Ontology annotations, and designed to generate both residue-level and sequence-level protein representation vectors. Specifically, it generates contextualized residue-level encodings that capture the biochemical context of individual amino acid residues, as well as a sequence-level encoding that summarizes the overall properties of the protein sequence. iFeature and Pfeature are comprehensive toolkits for generating various physicochemical encodings derived from protein sequences.

Based on these encoding strategies, we develop ProHL, a machine learning model that integrates multiple determinants influencing protein half-life to predict whether a query protein is long-lived or short-lived. The architecture of the ProHL model consists of three protein encoding modules and one classification module utilizing the output of the encoding modules for prediction (Fig. 2).

In the protein encoding modules, residue-level (Fig. 2A) and sequence-level encodings (Fig. 2B), as well as physicochemical encodings (Fig. 2C), are generated from protein sequences. Of the three encoding modules, 2D residue-level encodings (Fig. 2A) are projected into the same dimensional space (1D) as the sequence-level and physicochemical encodings for concatenation. For this projection, contextualized token embeddings produced by the ProteinBERT language model, which reflect sequence-dependent contextual information at each token position, were represented as tensors of size (embedding dimension × sequence length), and subsequently the encodings were projected into fixed-length 1D vectors for downstream classification task by using a CNN model since CNN instead of global mean pooling enables the generation of a fixed-length vectors that more effectively captures local contextual representations embedded in contextualized token embeddings [35]. The other two types of encodings (sequence-level encodings and physicochemical encodings) are then concatenated with the projected residue-level encodings, and the concatenated encodings are fed into the fully connected downstream neural network for prediction (Fig. 2D).

To determine appropriate model hyperparameters, we optimized eight key hyperparameters across the CNN and fully connected network: kernel size, number of filters, number of layers, number of units, dropout rate, learning rate, number of epochs, and batch size. To identify the optimal hyperparameter configuration, we performed a grid search combined with 10-fold cross-validation on the training dataset and selected the parameter set that achieved the highest MCC. Cross-validation was performed using the Adam optimizer with early stopping and a patience of 5 to prevent overfitting. The final hyperparameter configuration was as follows: a kernel size of 7×7, 8 filters, 2 layers, a number of units equal to the input feature dimension, a dropout rate of 0.2, and a learning rate of 10^-3^. The number of training epochs was determined to be 6 and the batch size was 32 based on the early stopping criterion.

**Fig. 3:**
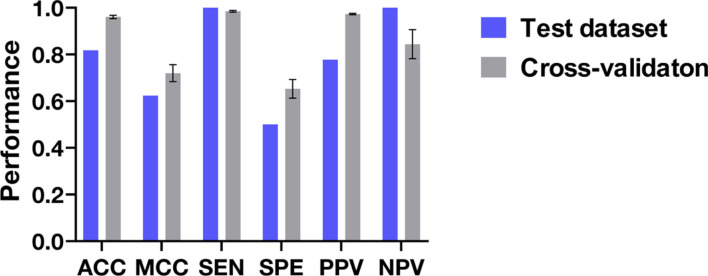
Performance of ProHL. Cross-validation performance is shown in gray and test performance is shown in blue. The standard errors were calculated from the 10-fold results. The x-axis represents the following performance metrics: accuracy (ACC), Matthews correlation coefficient (MCC), sensitivity (SEN), specificity (SPE), positive predictive value (PPV), and negative predictive value (NPV).

In the 10-fold cross-validation, the optimal model achieved an ACC of 0.961, PPV of 0.973, NPV of 0.844, SEN of 0.985, SPE of 0.653, and MCC of 0.720 (Fig. 3). The proposed model, designated ProHL, was further evaluated using an independent *E. coli* test dataset, comprising 7 long-lived and 4 short-lived proteins curated from existing literature. Although the limited sample size of the test dataset may not fully represent the generalizable performance of ProHL, it should be emphasized that the initial dataset comprised 1,149 proteins, representing 45% of experimentally quantifiable *E. coli* protein-coding sequences for which half-life data could be obtained[44]. Consequently, these 11 proteins represent the exhaustive set of experimentally validated samples that met our stringent inclusion criteria. ProHL achieved an ACC of 0.818, PPV of 0.778, NPV of 1.000, SEN of 1.000, SPE of 0.500, and MCC of 0.624 (Fig. 3). Despite the substantial class imbalance between long-lived and short-lived proteins in the training data, ProHL exhibited robust predictive performance, as evidenced by its high MCC. This indicates that ProHL maintained a balanced correlation between predicted and true labels without bias toward the majority class, suggesting effective capture of discriminative sequence and physicochemical determinants associated with protein half-life.

### Sub-Group Level Ablation Analysis of Physicochemical Encodings

In this study, various physicochemical and sequence-derived encodings were incorporated to capture the multifaceted determinants of protein half-life. In bacteria, protein degradation is primarily governed by proteolytic systems, such as ClpXP and Lon, which recognize substrates based on specific sequence and structural determinants, including degron-like motifs and conformational accessibility [45, 46]. While protein language model-based encodings capture rich contextual information, their implicit nature often limits direct biological interpretation. In contrast, physicochemical encodings offer explicitly defined descriptors that enable a more systematic analysis of the sequence-derived properties underlying protein stability.

To evaluate the relative contribution of these features, we categorized them into four biologically interpretable groups: (i) gapped *k*-mer-based composition descriptors (*e.g.*, Composition of *k*-spaced Amino Acid Pair), reflecting local residue interactions and protease recognition signals; (ii) Statistical and grouped composition descriptors (*e.g.*, Dipeptide Deviation from Expected Mean), representing global amino acid biases; (iii) physicochemical property-based descriptors (*e.g.*, Composition/Transition/Distribution), influencing folding stability and the exposure of degradation-prone regions; and (iv) sequence-order and autocorrelation descriptors (*e.g.*, Moran correlation, Sequence-Order-Coupling Number), encoding the spatial organization of physicochemical properties along the polypeptide chain.

**Fig. 4:**
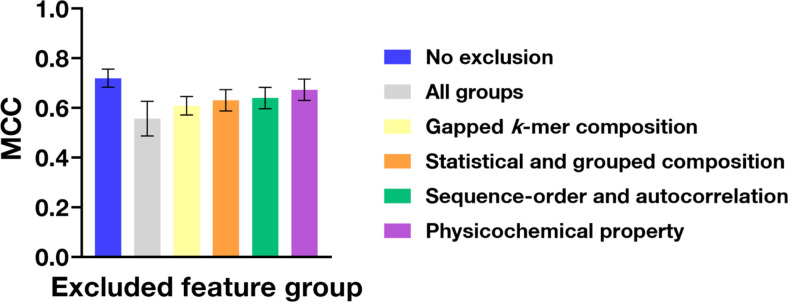
Sub-group level ablation analysis of physicochemical encodings. The contribution of each feature category to the predictive performance of ProHL was evaluated using the MCC. "No exclusion" represents the complete model, while “All groups” indicates the model excluding all four physicochemical feature categories. The categories evaluated include gapped *k*-mer-based composition, composition-derived descriptors, sequence-order and autocorrelation descriptors, and physicochemical property-based descriptors. Error bars represent the standard error calculated from 10-fold cross-validation. The performance decline observed upon the exclusion of individual groups underscores their relative importance and synergistic contribution to model accuracy.

We performed a feature-group-level ablation analysis using a leave-one-group-out strategy to assess the impact of each group on the model's MCC. Specifically, each feature group was systematically removed from the input while keeping all other features unchanged, and the resulting change in model performance was assessed. The baseline ProHL model achieved an MCC of 0.72. Notably, the removal of the *k*-mer-based composition group resulted in the most significant performance decline (MCC = 0.609), suggesting that local residue patterns and motif-like features are critical determinants of proteolytic susceptibility. The removal of Statistical and grouped composition-based and sequence-order-based group also led to substantial decreases in MCC (0.631 and 0.640, respectively), indicating that both global compositional bias and the spatial distribution of residues are essential for accurate stability prediction. Physicochemical property-based group exhibited a relatively smaller contribution (MCC = 0.673), potentially due to an overlap with representations captured by sequence-based encodings. Importantly, the simultaneous exclusion of all groups led to a pronounced decrease in MCC to 0.557 (Fig. 4), demonstrating that these explicit descriptors provide vital complementary information beyond the implicit representations learned by protein language models. Collectively, these findings highlight the synergistic effect of integrating multi-level sequence encodings and support the biological relevance of the selected physicochemical features in predicting protein half-life.

### Application to Identify a Bottleneck Enzyme in a Synthetic Pathway

In synthetic metabolic pathways composed of multiple recombinant enzymes, certain enzymes may act as a rate-limiting enzyme due to their insufficient cellular abundance, which limits the overall metabolic flux of the pathway and thereby creates a metabolic bottleneck [47]. Although several computational models have been developed to predict gene expression levels based on promoter strength, untranslated regions, and coding sequence features [22, 48], these approaches do not account for post-translational factors, such as protein homeostasis including half-life, which ultimately determine the levels of functional enzymes inside the cell.

**Fig. 5:**
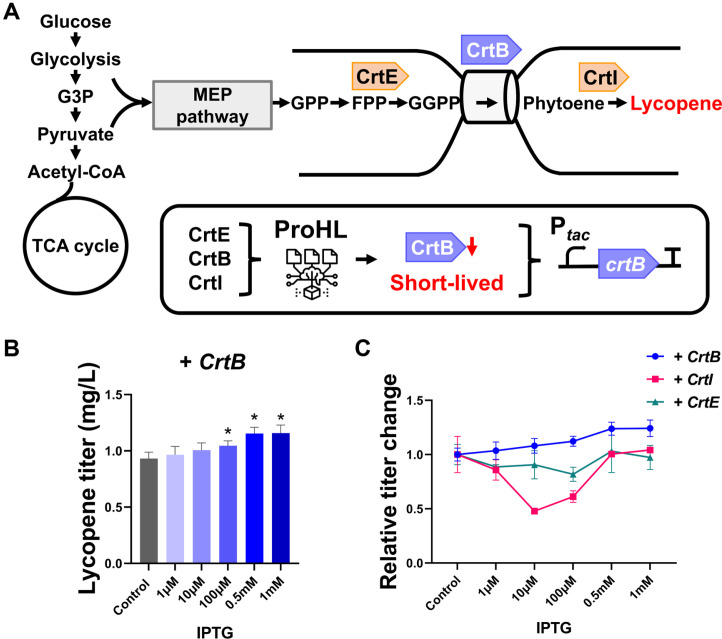
Evaluation of lycopene production in *E. coli* MG1655 with additional *crtB* expression. (**A**) The Lycopene biosynthetic pathway. The *crtE*, *crtB*, *crtI* genes were introduced to produce lycopene from FPP. The protein half-lives of CrtE, CrtB, and CrtI were predicted using ProHL, and CrtB was predicted as relatively short-lived. To compensate for its deficiency, additional The *crtB* was additionally expressed to enhance lycopene production. (**B**) Lycopene titers produced from engineered strain with different additional expression levels of *crtB* by inducing at different IPTG concentrations. (**C**) Relative fold changes in lycopene production upon individual overexpression of *crtB*, *crtI*, and *crtE* at various IPTG concentrations. The lycopene titers were normalized to those of the control strain. Asterisk (*) denotes *p*-value < 0.05. Error bars indicate standard deviations from three experiments.

To demonstrate the practical utility of our ProHL model, we applied it to identify short-lived enzymes within the lycopene biosynthetic pathway, which comprises three key enzymes: CrtB, CrtE, and CrtI (Fig. 5A). These enzymes catalyze the sequential conversion of farnesyl diphosphate (FPP) to lycopene [26].

Lycopene has garnered significant industrial interest owing to its strong antioxidant, anti-inflammatory, and anti-cancer properties, making it highly valuable in the food, feed, cosmetic, and pharmaceutical sectors [49, 50]. Metabolic engineering of microbial lycopene production has emerged as a sustainable alternative to conventional chemical synthesis [51, 52]. To date, extensive efforts have focused on increasing lycopene titer by manipulating enzyme genes to enhance flux toward lycopene and reduce diversion to competing pathways [26, 53, 54]. However, enzyme half-life has been largely overlooked as a critical determinant of pathway efficiency, primarily due to the difficulty of its measurement and the lack of computational prediction methods.

Among the three key enzymes in the lycopene biosynthetic pathway, our model predicted that CrtB has a relatively short half-life and may serve as a rate-limiting enzyme (Fig. 5A). To experimentally validate this prediction, we introduced an additional copy of the *crtB* gene under the control of a *tac* promoter on a separate plasmid and transformed it into the lycopene-producing *E. coli* strain [26]. The expression level of the additional *crtB* gene was modulated using Isopropyl β-D-thiogalactoside (IPTG) to assess its impact on lycopene biosynthesis. As shown in Fig. 5B, lycopene production steadily increased with higher IPTG induction. Specifically, lycopene titers were enhanced by approximately 24% when 0.5 mM IPTG was added. Interestingly, even with the addition of 1 mM IPTG, the lycopene titer did not significantly increase, similar to the result obtained with 0.5 mM IPTG. This suggests that CrtB expression was already sufficient for lycopene production at 0.5 mM IPTG.

The lycopene titer exhibited a dose-dependent increase at lower IPTG concentrations, but reached a plateau at higher induction levels (Fig. 5C). This observation suggests that while CrtB is a key bottleneck, its impact on the pathway flux may diminish once a certain threshold of expression is achieved. Specifically, the lack of further improvement at higher IPTG concentrations could indicate that the stability-induced bottleneck of CrtB was sufficiently resolved, shifting the rate-limiting step to other parts of the metabolic network. Alternatively, the plateau may reflect the onset of a metabolic burden, where the physiological stress and resource competition associated with high-level protein expression outweigh the gains in enzyme activity. In this context, the final metabolite titer is likely determined by a complex interplay between the targeted resolution of enzyme-specific constraints and the inherent physiological limits of the host strain under strong induction.

To further validate that CrtB is the only rate-limiting bottleneck in the lycopene biosynthetic pathway, we individually overexpressed CrtI and CrtE. In contrast to CrtB, the additional expression of these enzymes resulted in lycopene fold changes that were either similar to or lower than those of the control (Fig. 5C). These observations suggest that CrtI and CrtE do not function as rate-limiting enzymes in this pathway; instead, their overexpression may impose an unintended metabolic burden, potentially reducing the lycopene titer. Collectively, these results demonstrate that ProHL can effectively identify short-lived enzymes that function as metabolic bottlenecks, thereby providing a practical strategy to guide targeted pathway optimization and improve metabolite production. It should be noted that the current validation of ProHL relies on functional metabolic outcomes rather than direct measurements of protein degradation rates. Direct quantification of *in vivo* protein stability remains technically challenging, as the introduction of detection tags—such as His-tag or GFP—can impose structural modifications that interfere with a protein’s intrinsic stability and metabolic turnover [55]. Such exogenous factors may lead to experimental artifacts that confound the validation of half-life predictions for native enzymes. Therefore, in this study, we employed lycopene production as a functional surrogate to validate the practical utility of ProHL’s predictions. The selective improvement in titer observed only with the predicted short-lived enzyme (CrtB), but not with CrtI or CrtE, suggests that ProHL can effectively identify stability-induced bottlenecks relevant to actual metabolic flux, even in the absence of direct turnover assays. This positioning establishes the lycopene production experiment as a robust proof-of-concept for ProHL-guided targeted pathway optimization.

### ProHL Web Server

**Fig. 6:**
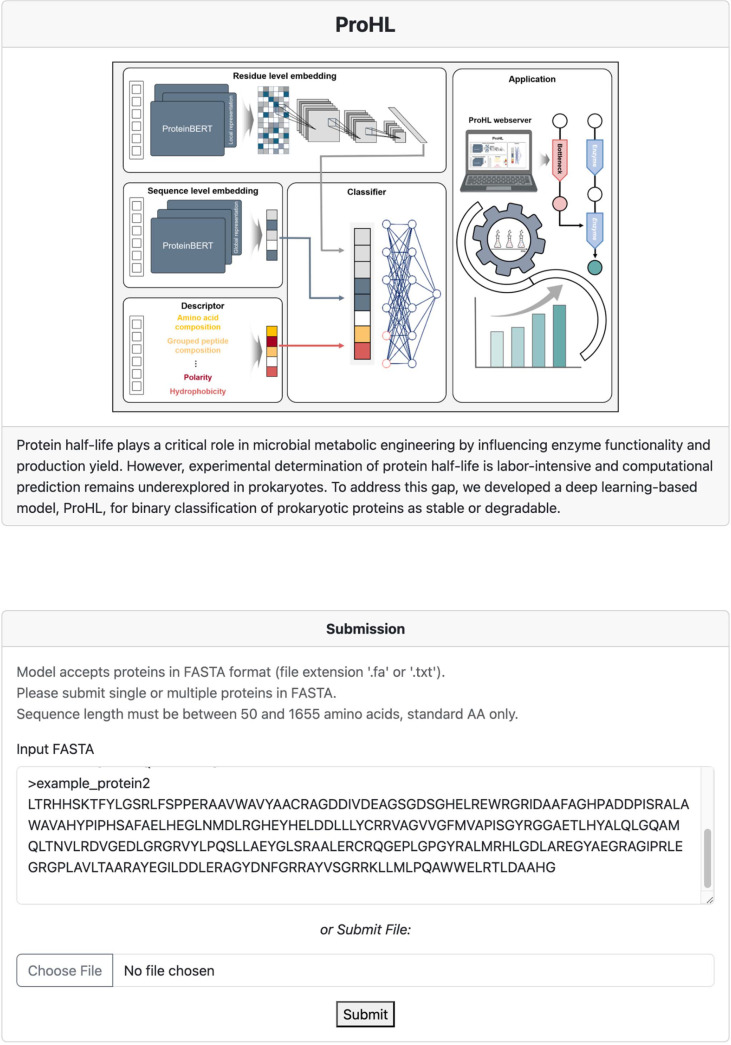
Web server of ProHL. The ProHL web server provides an intuitive interface for predicting whether a query protein sequence is long-lived or short-lived. Users can input single or multiple sequences by pasting them into the text box or uploading a FASTA file. Prediction results can be downloaded as an excel file containing the predicted half-life classification for each protein.

ProHL, along with the associated training and test datasets utilized in this study, is publicly accessible at our web server, http://prohl.lile.bio. Users can predict whether a query protein is long-lived or short-lived in *E. coli* (Fig. 6). A query sequence must consist of standard amino acids and has a length of 50 to 1655 amino acids. Upon submission, the web server automatically performs feature computation and all necessary preprocessing steps, and then predicts whether the protein is short-lived or long-lived.asdasd

### Limitation of ProHL

In this study, we categorized enzymes as “short-lived” based on an absolute half-life threshold of 1 hours, facilitating a targeted strategy to alleviate stability-induced bottlenecks. However, absolute protein half-life values can vary significantly depending on the physiological state and nutrient availability [44]. However, we hypothesized that proteins with a rapid turnover rate are largely governed by their intrinsic sequence features and structural motifs—such as specific degrons or proteolytic recognition sites—which remain relatively consistent regardless of the environment [45, 56]. While the absolute half-life of a protein may shift across different conditions, its relative stability ranking compared with other proteins is likely to be preserved. This is precisely why ProHL was designed as a categorical classifier (short-lived vs. long-lived) rather than a regression model for absolute values; a categorical approach is more robust to the inherent noise and variability of absolute half-life measurements across diverse conditions.

Nevertheless, as the model is trained on data derived from a single species, its predictive performance across diverse bacterial species may be constrained by differences in proteostasis mechanisms and cellular physiology. Despite these limitations, organism-specific models remain highly valuable in practice, particularly for well-established industrial hosts such as *E. coli*, where they can directly support strain optimization. As more experimental datasets become available across diverse bacterial species and growth conditions, future work will focus on systematically addressing these sources of variability. In particular, fine-tuning and transfer learning approaches will enable the adaptation of ProHL to new biological contexts while preserving the core sequence-derived representations learned from existing data. By extending the model to account for both species-specific and condition-dependent effects, ProHL can evolve toward a more generalizable framework for predicting protein turnover across diverse biological systems. Ultimately, ProHL provides not only a robust computational tool but also a perspective shift toward a new strategy for overcoming metabolic bottlenecks in microbial cell factory construction.

## Conclusion

This study highlights the importance of protein half-life as a design parameter in metabolic engineering and demonstrates the potential of computational models such as ProHL to guide pathway optimization. By moving beyond conventional strategies focused solely on transcriptional and translational control to proteostasis, the integration of proteostasis-aware approaches offers new opportunities to fine-tune synthetic pathways and accelerates the construction of microbial cell factories. The accessible model, curated datasets, and inference pipeline enable rapid screening and design of enzyme variants with improved intracellular half-life stability, thereby supporting rational strain optimization and synthetic biology–driven pathway enhancement. Current limitations, such as the relatively small size of bacterial half-life datasets and species-specific variability, indicate that further development of larger and more diverse training sets, as well as incorporation of structural and systems-level features, will be critical for broader applicability.

## Supplemental Materials

Supplementary data for this paper are available on-line only at http://jmb.or.kr.
