# Corrigendum to: Deep Learning-Based Protein Half Life Prediction for Identifying Rate Limiting Enzymes in Metabolic Pathways to Alleviate Bottleneck Reactions

**DOI:** 10.4014/jmb.2026.3602.C02

**Published:** 2026-05-13

**Authors:** Yunhyeok Lee, Jun Ren, Jingyu Lee, Minh Thi-Hong Tran, Yubin Kim, Youngseo Chang, So Hee Oh, Hyang-Mi Lee, Dokyun Na

**Affiliations:** Department of Biomedical Engineering, Chung-Ang University, Seoul 06974, Republic of Korea

## Abstract

In the article titled “Deep Learning-Based Protein Half-Life Prediction for Identifying Rate Limiting Enzymes in Metabolic Pathways to Alleviate Bottleneck Reactions,” we would like to address an authorship error that occurred during the revision process. So Hee Oh was inadvertently included in the author list due to a clerical error. We have therefore updated the author list to accurately reflect the contributors to this research.

The corrected author list is as follows:

Yunhyeok Lee^†^, Jun Ren^†^, Jingyu Lee, Minh Thi-Hong Tran, Yubin Kim, Youngseo Chang, Hyang-Mi Lee^*^, and Dokyun Na^*^

We acknowledge this correction and confirm that it does not affect the results, interpretations, or conclusions of the article.

The authors would like to apologies for any inconvenience caused. (http://jmb.or.kr)

