# Supplementary material for: Deep Learning-Based Protein Half-Life Prediction for Identifying Rate-Limiting Enzymes in Metabolic Pathways to Alleviate Bottleneck Reactions
Source: J Microbiol Biotechnol. 2026 Apr 27;36:e2601071. doi: 10.4014/jmb.2601.01071 (PMC13128668; doi:10.4014/jmb.2601.01071)
Supplement: Supplementary file 1 [file jmb-36-e2601071-supple.pdf]

Submit to Journal of Microbiology and Biotechnology

Deep Learning-Based Protein Half-Life Prediction for Identifying Rate-Limiting Enzymes in Metabolic Pathways to Alleviate Bottleneck Reactions

Yunhyeok Lee†, Jun Ren†, Jingyu Lee, Minh Thi-Hong Tran, Yubin Kim, Youngseo Chang, So Hee Oh, Hyang-Mi Lee*, and Dokyun Na*

Department of Biomedical Engineering, Chung-Ang University, Seoul 06974, Republic of Korea

† These authors equally contributed to this study.

* Correspondence.

Dokyun Na, Ph.D. blisszen@cau.ac.kr, Tel: +82-2-820-5690

Hyang-Mi Lee, Ph.D: myhys84@gmail.com

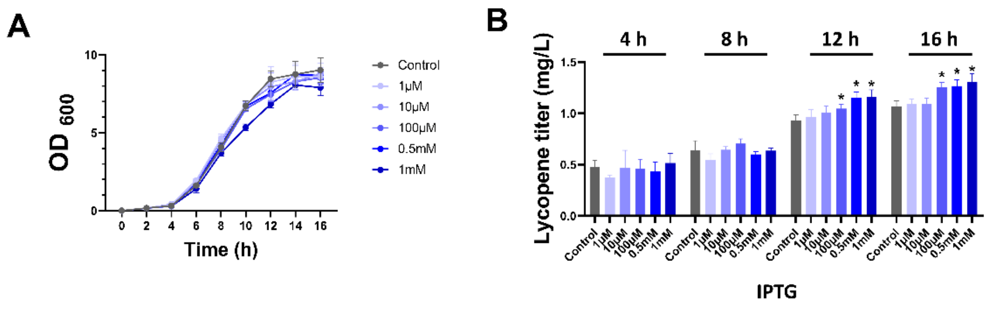

Fig. S1. Effect of additional *crtB* expression on *E. coli* growth and Lycopene titer at different time points.

In *E. coli* MG1655, *crtB* was additionally expressed at varying IPTG concentrations and co-expressed with plasmids pWA-IEB and pA-SRAI. Lycopene titers were measured at different time points to evaluate the effect of *crtB* overexpression on lycopene production dynamics. All experiments were performed in the dark, and samples were prepared in triplicate. Asterisk (*) denotes p-value < 0.05. Error bars indicate standard deviations.
